# Protective Effect of Oyster Peptides Derived From *Crassostrea gigas* on Intestinal Oxidative Damage Induced by Cyclophosphamide in Mice Mediated Through Nrf2-Keap1 Signaling Pathway

**DOI:** 10.3389/fnut.2022.888960

**Published:** 2022-05-16

**Authors:** Hui Chen, Huizhen Zheng, Tiejun Li, Qihong Jiang, Shulai Liu, Xuxia Zhou, Yuting Ding, Xingwei Xiang

**Affiliations:** ^1^College of Food Science and Technology, Zhejiang University of Technology, Hangzhou, China; ^2^Key Laboratory of Marine Fishery Resources Exploitment and Utilization of Zhejiang Province, Zhejiang University of Technology, Hangzhou, China; ^3^National Engineering Research Center of Seafood, Collaborative Innovation Center of Seafood Deep Processing, School of Food Science and Technology, Dalian Polytechnic University, Dalian, China; ^4^Zhejiang Marine Fisheries Research Institute, Zhoushan, China

**Keywords:** oyster peptides, cyclophosphamide, oxidative stress, intestinal mucosa, Nrf2-Keap1 antioxidant pathway

## Abstract

Oyster peptide (OP) has exhibited useful biological activities and can be used in multi-functional foods. OP has been reported to play a significant role in intestinal protection, but its specific mechanism is still not completely understood. The aim of this study was to analyze the potential effect of OP on oxidative damage of mice intestine induced by cyclophosphamide (Cy). The experimental results revealed that intragastric administration of OP significantly increased average bodyweight, improved ileum tissue morphology and villus structure, as well as increased the activities of superoxide dismutase (SOD), catalase (CAT) and glutathione peroxidase (GSH-Px) in oxidized mice serum and liver. The content of malondialdehyde (MDA) in the mice serum and liver homogenate was found to be markedly decreased. Moreover, OP significantly increased the relative mRNA expression levels of superoxide dismutase (SOD), glutathione peroxidase (GSH-P_X_), quinone oxidoreductase (NQO1) and heme oxidase-1 (HO-1) in ileum. Western-blot results indicated that prior administration of OP significantly up-regulated the Nrf2 production in ileum, and substantially decreased then Keap1 gene expression. In conclusion, intake of OP was found to markedly improve intestinal oxidative stress *in vivo*, and this effect was primarily mediated through the simulation of antioxidant Nrf2-Keap1 signaling pathway. This study is beneficial to the application of peptide nutrients in the prevention or mitigation of intestinal oxidative damage.

## Introduction

Oxidative stress related injury refers to the imbalance between oxidative and anti-oxidative reactions caused by excessive production of reactive oxygen species (ROS) in the body (1). The various ROS species are primarily generated and exhibit their functions in mitochondria. These include superoxide anion radical, hydrogen peroxide (H_2_O_2_), alkane peroxide radical, lipid peroxide radical, nitric oxide (NO·) and hydroxyl radical (HO·) (2). At low concentrations, ROS generally plays a beneficial regulatory role through inducing apoptosis of injured or senescent cells and acting as the different mediators of cell signaling pathway regulation (3). However, excessive production of ROS can be harmful to the body, leading to significant oxidative stress and can substantially weaken intestinal mucosal immune barrier function (4). The intestine is a vital organ of the human body and the major site of digestion and absorption. As a biological barrier (5), it is frequently exposed to oxidative stress and other stress conditions, resulting in increased infiltration of the toxins. Excessive accumulation of oxygen free radicals can effectively destroy the gut mucosal barrier and disrupt intestinal flora, thus affecting the body's homeostasis system (6). Furthermore, oxidative stress can function as an important factor in intestinal inflammation. Accumulating evidences suggest that the use of antioxidants could be useful for the treatment of inflammatory bowel disease, intestinal mucosal infections, ulcerative colitis and colon cancer (7–9).

Cyclophosphamide (Cy) is an alkylating compound, which is often used as an antitumor agent and chemotherapeutic agent (10, 11). It mainly acts through causing cross-linking in DNA, blocking DNA replication as well as synthesis, and thus preventing cell proliferation. The effects of Cy have been found to be non-specific and it can not only eliminate the malignant cells, but at the same time can also damage the various normal cells (such as lymphocytes, gastrointestinal mucosa cells, etc.) (12). It can also disrupt the normal structure of gastrointestinal mucosa and adversely affect intestinal mucosa thereby causing immune disorders and severe side-effects. Extensive use of Cy can cause nausea and vomiting, diarrhea, intestinal barrier destruction, increase intestinal permeability, gastrointestinal mucosal damage, enhance exposure to immune deficiency and secondary infection, oxidative stress, intestinal microflora structural disorders and other side effects (13). Thus, in this study, Cy was used as an inducer to establish a model of intestinal oxidative damage in mice.

Antioxidants present in the food can significantly improve the oxidative state of the body and help to maintain the oxidative/antioxidant balance in the gut (14, 15). The use of food proteins and their constituent peptides which exhibit relatively fewer side effects as antioxidants can serve as an effective approach. Oyster is one of the largest shellfish varieties found in the world. It is extremely rich in different nutrients, including the trace elements, proteins, fatty acids, glycogen, vitamins and taurine. Oyster, as the largest shellfish cultured in the world, is rich in various marine based active peptides, which has become a research hotspot at home and abroad (16, 17). The Marine oyster peptides (OP) possess specific amino acid compositions and sequences, which are quite different from those of the terrestrial proteins. There may be some peptides with specific biological activities. In recent years, a number of studies have reported that oyster active peptide can display multifarious biological activities, such as those related to anti-oxidation (18), lowering of blood pressure (19), inhibition of tumor cancer (20), improving learning and memory (21), and enhancing immunity (22). In addition, a few studies have found that the peptides obtained from the various substances can also exhibit potent antioxidant properties. For example, the antioxidant peptide (ATVY) obtained by Yang et al. from the hydrolyzed protein of black shark skin has displayed good free radical scavenging activity and can be used for the production of antioxidant food additives (23). In addition, the purified peptides obtained from the extract of *Marphysa sanguinea* have demonstrated significant antioxidant activity and reduced the contents of catalase (CAT), superoxide dismutase (SOD), glutathione peroxidase (GSH-PX) and malondialdehyde (MDA) (24). It also inhibited the abnormal secretion of proinflammatory cytokines such as nitric oxide (NO), inducible nitric oxide synthase (iNOS) and cyclooxygenase 2 (COX-2). In another study, Mirzapour-kouhdasht et al. prepared gelatin peptides from fish by-products which displayed potent free radical scavenging effects and pronounced antioxidant effects after gastrointestinal digestion *in vitro* (25). In addition, the small molecule peptides obtained from ALI have exhibited strong DPPH (26), hydroxyl radical and superoxide radical scavenging activities, iron reducing antioxidant and metal chelating properties, and inhibited enzymatic activities of ACE, α-amylase and glucosidase. However, there are only few studies which have examined the effect of OP on oxidative damage of intestinal mucosa induced by Cy.

Here, the potential protective effects of OP on intestinal mucosa were explored by establishing a oxidative injury mouse model of intestinal mucosa, and the underlying mechanism was elucidated. As the advance and the novelty, this study analyzed the relationship between antioxidative peptide and Nrf2-Keap1 pathway *in vivo*. The findings of this study might provide evidence for further understanding the protective mechanism of OP on intestinal mucosal injury, and thus provide a better natural alternative health food both for the prevention and treatment of oxidative stress injury.

## Materials and Methods

### Experimental Animals

Male SPF BALB/C mice aged 5–7 weeks and weighing 15–18 g were used in the present study. The mice were purchased from Slack Laboratory Animal Co., LTD. (Shanghai, China), and the license number was: SCXK (Shanghai) 2017-0005. Before the formal beginning of the experiments, the mice were placed in the animal room for adaptive feeding for 7 days, during which they were free to ingest food and water. Environmental conditions were set as: temperature 25 ± 1°C, humidity 50 ± 3%, light and dark cycle for 12 h. This study was carried out by the guidelines of the Animal Welfare Act and the Guide for the Care and Use of Laboratory Animals, which is approved by the Animal Ethics Committee of Zhejiang University of Technology (20210308038).

### Chemicals and Reagents

Cyclophosphamide was obtained from Aladdin Chemical Co., LTD (Shanghai, China). Catalase (CAT), superoxide dismutase (SOD), glutathione peroxidase (GSH-PX), and malondialdehyde (MDA) detection kits were purchased from Nanjing Jianguo Institute of Biological Engineering (Nanjing, China). Antibodies against Nrf2 and Keap1 were obtained from Abcam (Cambridge, UK). In addition, monoclonal antibody against β-actin was purchased from Santa Cruz Biotechnology, Inc. (Santa Cruz, CA, USA). All other reagents obtained were commercially available and of analytical grade.

### Management of Animals

The acclimated mice were randomly divided into four different groups according to body weight (*n* = 8): control group (C), Cy model group (Y), OP high-dose group (HP) and low-dose group (LP). Mice were intragastrically administered OP for 21 successive days, and intraperitoneal Cy was injected for 3 days (days 18–21). The dose of Cy was determined according to our previous research, as shown in Table 1. The body weight and the vital physical signs of the mice were examined and recorded daily. At the end of the experiment, all the mice were sacrificed by cervical dislocation. The cardiac blood samples were collected at 2,000 r/min and centrifuged for 10 min, −80 °C. The samples of thymus, spleen, liver, ileum, serum and feces were obtained and stored at −80°C for further experiment, respectively.

**Table 1:** Experimental grouping of mice.

**Groups**	**Oral administration** **(days 1–21)**	**Intraperitoneal injection** **(days 18, 19, 20, 21)**
C	Saline	Saline
Y	Saline	50 mg Cy /kg BW/day
LP	200 mg OP/kg BW/day	50 mg Cy /kg BW/day
HP	400 mg OP/kg BW/day	50 mg Cy /kg BW/day

### Determination of Average Daily Gain in Mice

On the 21st day of the experiment, the organ index of the mice after the death was determined by using the following formula:

Organ Index = organ weight (mg)/body weight (g) × 10

### Hematoxylin-Eosin Staining of Ileum of Mice

The fresh ileum of mice was rinsed several times with the normal saline and placed in a clean eppendorf tube. The ileum was fixed in 10 mL 4% paraformaldehyde at room temperature for 24 h. Thereafter, ethanol dehydration, xylene transparent and paraffin embedding were carried out, respectively. Finally, the paraffin blocks were continuously cut by a slicer to obtain intestinal sections with a thickness of approximately 6 μm. Three sections of each tissue block were randomly selected for hematoxylin-eosin (HE) staining (27), and the histopathological changes of the intestine were observed under the microscope (Olympus CX23, Japan).

### Electron Microscopic Observation

The ileum was removed after mouse the dissection, washed with PBS solution, and dried with filter article. A section of the empty intestine was obtained and fixed in a centrifuge tube containing glutaraldehyde. Transmission electron microscope (TEM) samples were treated as follows: The ileum of mice was washed several times with PBS solution and fixed in 1% osmium solution for 1 h. After rinsing with the distilled water for three times, the ileum was dehydrated with gradient ethanol and pure acetone successively. The ileum tissue was placed overnight in Spurr resin and polymerized at the temperature of 70°C. Thereafter, the intestine was cut into slices and then stained with uranium acetate and lead citrate. The ileum tissue was observed by TEM and SEM photographed (28). The sample was dehydrated with the gradient ethanol and embedded in the sample section. The samples were dried with liquid carbon dioxide critical point. The ileum surface was coated with a layer of metal composite material with a thickness of about 30 nm by ion sputtering.

### Detection of Antioxidant Enzyme Content in the Serum of Mice

The mice were anesthetized and sacrificed by neck amputation, followed by blood collection. The blood was subjected to the centrifugation at 4000 r/min and incubated at 4°C for 15 min. SOD, GSH-Px, CAT and MDA antioxidant kits were used to detect the contents of the various antioxidant factors in the serum of different groups of mice, according to the manufacturer's instructions.

### Determination of Antioxidant Factors in Mouse Liver Tissue Homogenate

The mouse livers were weighed to about 100 mg and thoroughly ground in a tissue homogenizer. The supernatant was obtained after centrifugation at 3000 r/min for 10 min and stored at the temperature of 4°C. The contents of antioxidant enzymes SOD, GSH-Px, CAT and MDA were detected on the basis of the manufacturer's instructions of the commercial antioxidant kits.

### Quantitative Real-Time Polymerase Chain Reaction

The relative mRNA expression levels of GSH-PX, SOD, CAT, NQO1 and HO-1 were detected through RT-PCR. The total RNA of the sample was extracted from the ileum by using 1 mL ice-cold TRIzol reagent (Ambion, Carlsbad, USA). The mass and concentrations of extracted RNA were determined via spectrophotometry at 260 and 280 nm, respectively. The first strand of cDNA was synthesized by using a Prime-Script 1st Strand cDNA Synthesis Kit (Takara, Dalian, Japan). The different primer sequences were designed by Primer 5 software and synthesized by Shanghai Shenggong Biotechnology Co., Ltd. (Shanghai, China), the details of which have been shown in Table 2. The qRT-PCR was performed by using the Applied Biosystems ViiA™7 Real-Time PCR system. Thereafter, PCR reaction was carried out according to the standard operation process of SYBR Green qPCR test kit. The RT-PCR reaction was carried out based on the following procedure: pre-denaturation at the temperature of 95°C for 30 s, followed by 40 cycles of the conditions: 95°C for 5 s and 65°C for 34 s. The cyclic threshold (CT) values of each gene were normalized using β-actin. With β-actin as internal reference gene, the relative mRNA expression level of target gene was calculated according to the 2^−ΔΔCt^ method (29). The experiment was repeated in triplicate.

**Table 2:** RT-PCR primer sequences.

**Gene**	**Gene accession number**	**Primer sequence 5^**′**^-3^**′**^**	**Product size (bp)**
SOD	NM_011434.1	F: ATGGCGATGAAAGCGGTGTG R: TTACTGCGCAATCCCAATCACTC	465
GPX	NM_ 008160.6	F: GCAATCAGTTCGGACACCAG R: CACCATTCACTTCGCACTTCTC	126
HO-1	NM_010442.2	F: GATAGAGCGCAACAAGCAGAA R: CAGTGAGGCCCATACCAGAAG	111
NQO1	NM_008706.5	F: GGACATGAACGTCATTCTCT R: TTCTTCTTCTGCTCCTCTTG	261
β-actin	NM_007393	F: AGTGTGACGTTGACATCCGT R: GCAGCTCAGTAACAGTCCGC	298

### Western Blot Analysis

The ileum lysates were prepared using the Radio Immunoprecipitation Assay (RIPA) lysate buffer. The protein concentrations of the lysates were analyzed through bicinchoninic acid (BCA) protein determination kit (Beyotime, Shanghai, China). Approximately 30 μg of proteins were isolated using SDS-PAGE (10%) solution and then transferred to a 0.45 μm polyvinylidene fluoride (PVDF) membrane (Merck Millipore, MA, USA). The membrane was then blocked with 5% skim milk and incubated with the primary antibody (Abcam, Cambridge, UK) at the temperature of 4°C overnight. The membranes were washed three times with TBS for 5 min. Thereafter, the membrane was incubated within HRP-conjugated secondary antibody at the room temperature for about 2 h and rinsed three times for 15 min. The chemiluminescence imaging was carried out using the protein using enhanced chemiluminescence (ECL) reagent (Beyotime, Shanghai, China) (30). Finally, Image J software was used to test and analyze the band density, and each sample was analyzed three times.

### Statistical Analysis

All experimental data was analyzed using SPSS17.0 software. The experimental data was expressed as mean ± standard error (SEM). One-way variance (ANOVA) and Tukey tests were adopted for the statistical significance analysis. *P* < 0.05 was considered to be statistically significant.

## Results and Discussion

### Effect of OP on Average Daily Gain in Mice

**Figure 1:**
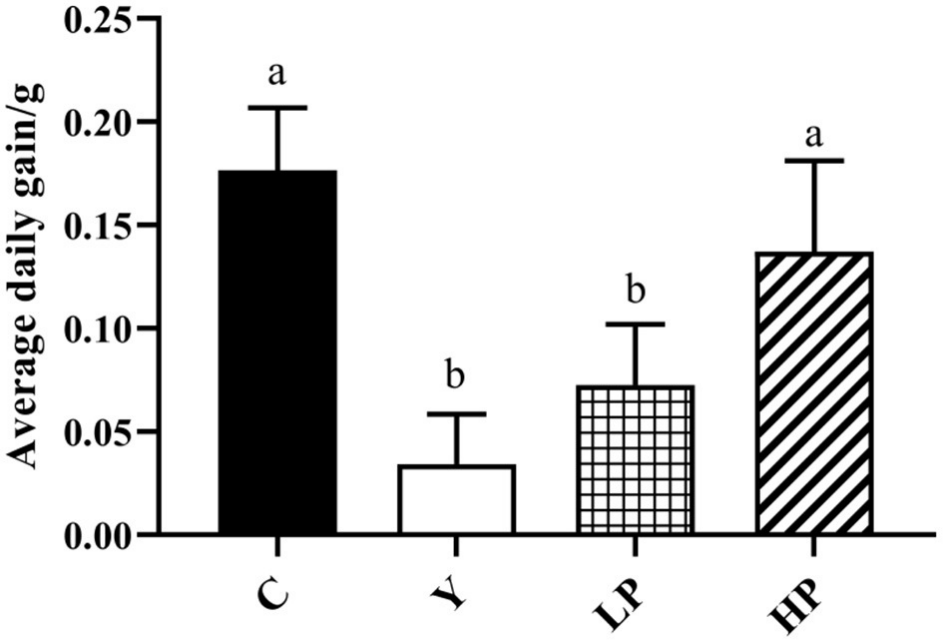
Effects of OP on the average daily gain in oxidative injured mice. Different letters in the figure represent significant differences (*P* < 0.05). C: the normal group; Y: the Cy-induced group; LP: Cy+200 mg/kg OP; HP: Cy+400 mg/kg OP.

The mice in Cy treated group showed significant stress reactions such as hair loss and luster, lethargy, slow movement and yellow feces on the second day after intraperitoneal injection of Cy, compared to the normal group (Figure 1). In addition, the average daily gain of mice after Cy injection was found to be decreased (*P* < 0.05). Compared with Cy group, early intragastric administration of high-dose OP significantly increased the average daily gain of mice (*P* < 0.05). However, the mice treated with low dose OP also significantly increased the average daily gain, but the effect was not found to be significant (*P* > 0.05).

### Effects of OP on Ileum Morphology in Mice

**Figure 2:**
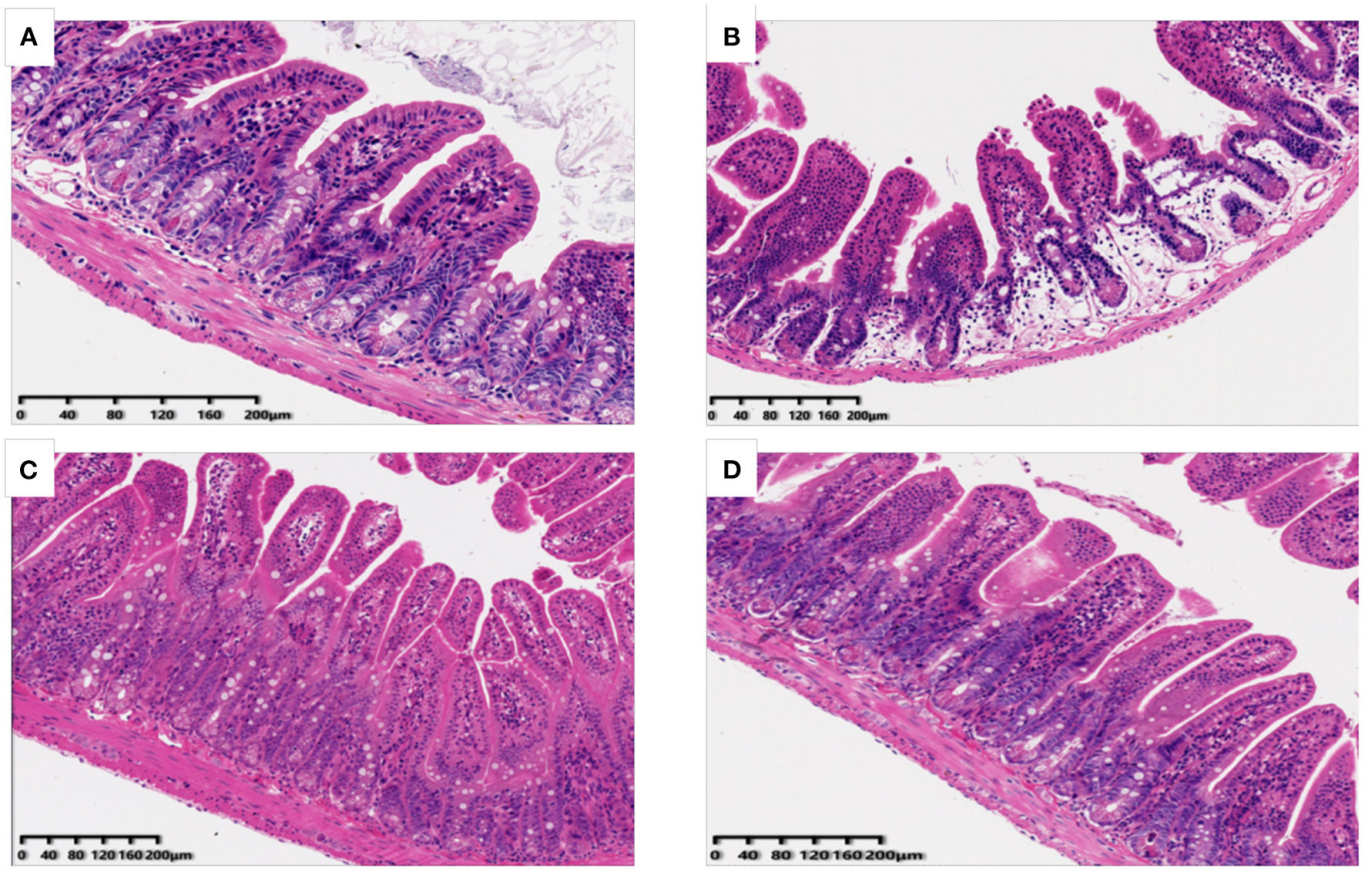
Effects of OP on ileum histomorphology in oxidative injured mice [**(A)**: Normal group; **(B)**: Cy-induced group; **(C)**: LP: Cy+200 mg/kg OP; **(D)**: HP Cy+400 mg/kg OP; 200x].

The potential effects of OP on ileum tissue morphology of mice were also analyzed and the results have been shown in Figure 2. It was found that in the normal group of mice ileum, villi was arranged orderly, appeared slender and compact with complete structure as well as thick intestinal wall. In the Cy treated group, the villi of ileum were observed to be loose and short, and some of the villi were broken and detached. After the OP pre-treatment, the length of villi in the LP and HP group was restored, and the structure was observed to be more complete as well as orderly, and the damaged condition was substantially improved. These results suggested that intake of OP can effectively protect ileum tissue of immunosuppressed mice to a certain extent, and reduce intestinal villi damage caused by Cy.

### Electron Microscopic Observation of Ileum in Mice

**Figure 3:**
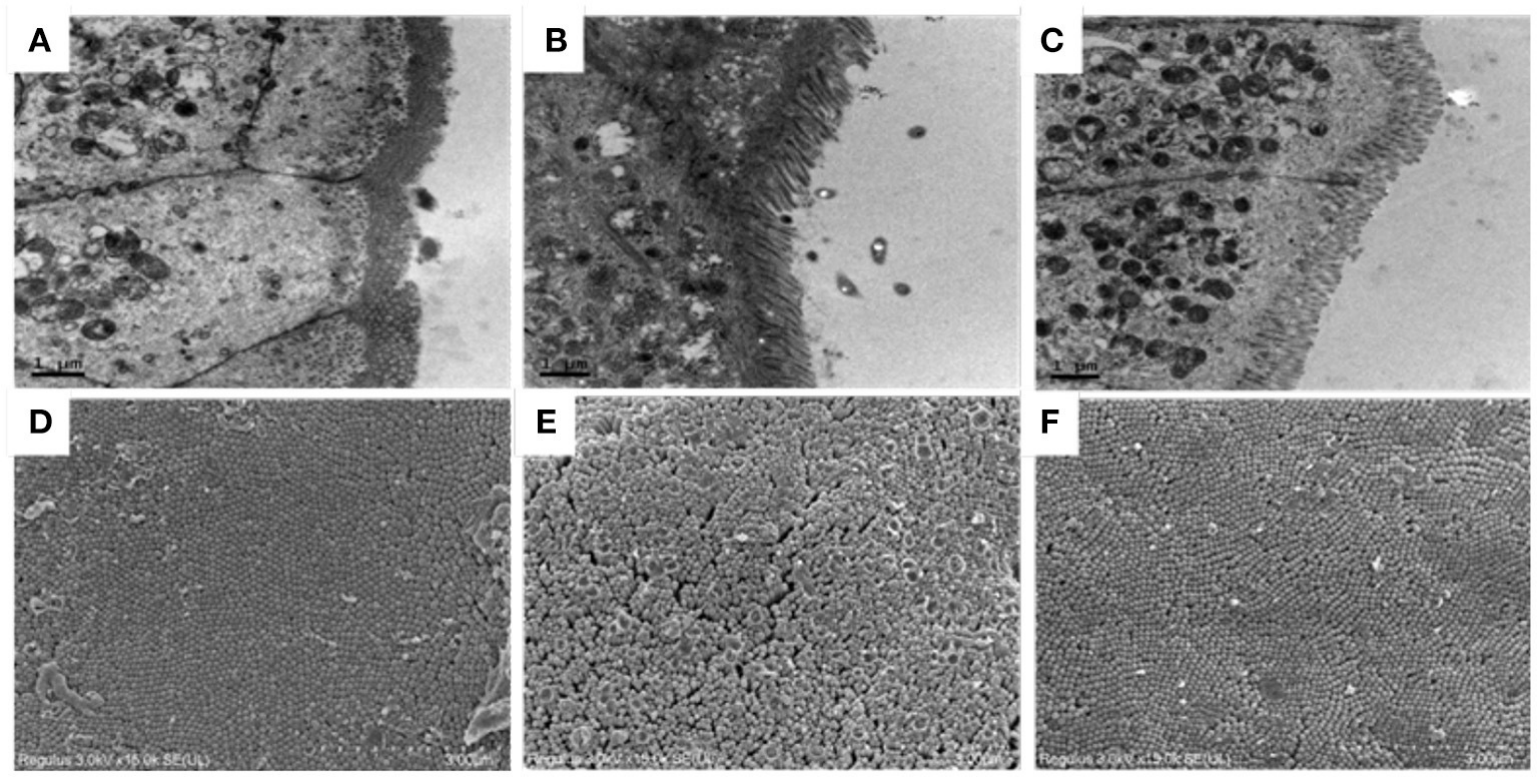
Effects of OP on ileum villi morphology in immunosuppressed mice. **(A–C)** Transmission electron microscopy, 10K×, **(A)**: Normal group, **(B)**: Cy-induced group, **(C)**: Cy+400 mg/kg OP, **(D–F)**: Scanning electron microscopy, 15K×, **(D)**: Normal group, **(E)**: Cy-induced group, and **(F)**: Cy+400 mg/kg OP.

TEM results of ileum of all mice have been shown in Figures 3A–C. In the normal group, the microvilli were found to be arranged and distributed in a very orderly manner, with long villi and almost no gap, and the close connection was clearly visible in the figure. However, compared with the normal group, Cy induced the intestinal microvilli of mice into rare and disordered state, with irregular tight connections, and the intestinal villi were observed to be severely damaged. After early intragastric administration of OP, the intestinal mucosa structure of the mice was noted to be significantly improved and the structure of microvilli was restored. The SEM results of ileum showed that the ileum structure of mice in the normal group was flat as well as smooth, and the microvilli were closely connected, in Figures 3D–F. The microvilli in the Cy model group were not consistent in height and were arranged irregularly, and some microvilli were found to be broken and fractured. The intestinal microvilli of mice treated with OP appeared to be relatively repaired, with small spaces between them as well as smooth surface, and almost returned to the normal state. These results indicated that intake of OP can markedly repair the damage of Cy induced ileum mucosa in mice.

### Effect of OP on Serum Antioxidant Enzyme Level in Mice

**Figure 4:**
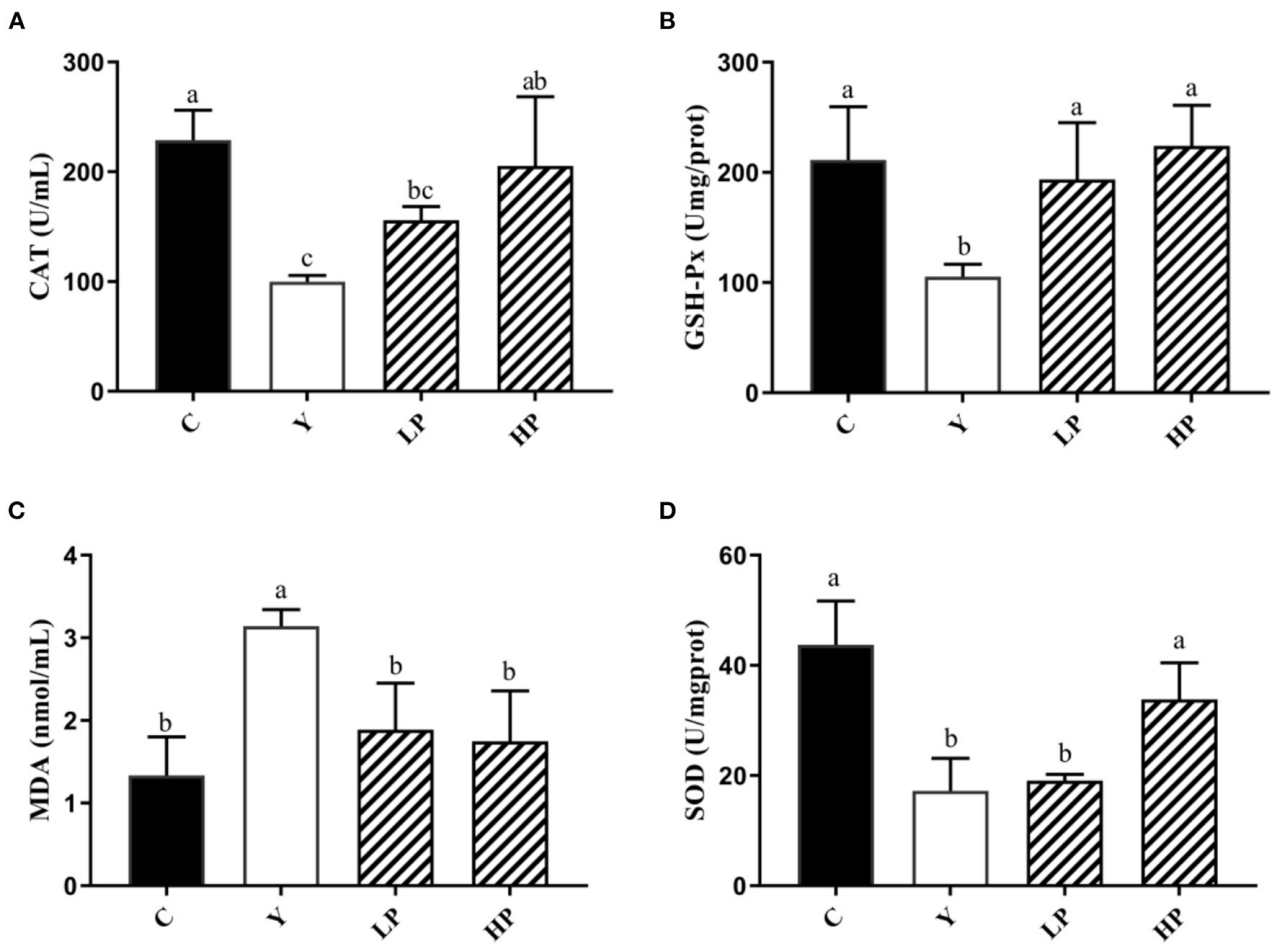
Effects of OP on the contents of CAT **(A)**, GSH-Px **(B)**, MDA **(C)**, and SOD **(D)** in serum of oxidative injured mice. Different letters in the figure represent significant differences (*P* < 0.05). C: the normal group; Y: the Cy-induced group; LP: Cy+200 mg/kg OP; HP: Cy+400 mg/kg OP.

We also investigated the dose dependent effects of OP on the content or activities of serum antioxidant enzyme in mice, as shown in Figure 4. The activities of SOD, CAT and GSH-Px in serum of Cy treated mice were distinctly decreased, and the activity of MDA was significantly increased (*P* < 0.05), compared with normal group. The intervention with high dose OP increased the contents of SOD, CAT and GSH-Px in serum of mice (*P* < 0.05), but significantly decreased the activity of MDA (*P* < 0.05), compared with the Cy group. Interestingly, low dose of OP intake also increased GSH-Px activity of serum in mice (*P* < 0.05), and decreased the content of MDA in the serum (*P* < 0.05), but did not display significant effect on the activities of SOD and CAT in the serum (*P* > 0.05). These findings suggested that the prior administration of OP boosted the antioxidant capacity *in vivo*, thereby modulating the oxidative stress response caused by Cy even in a dose-dependent manner.

### Effect of OP on Antioxidant Enzyme Level in Mouse Liver

**Figure 5:**
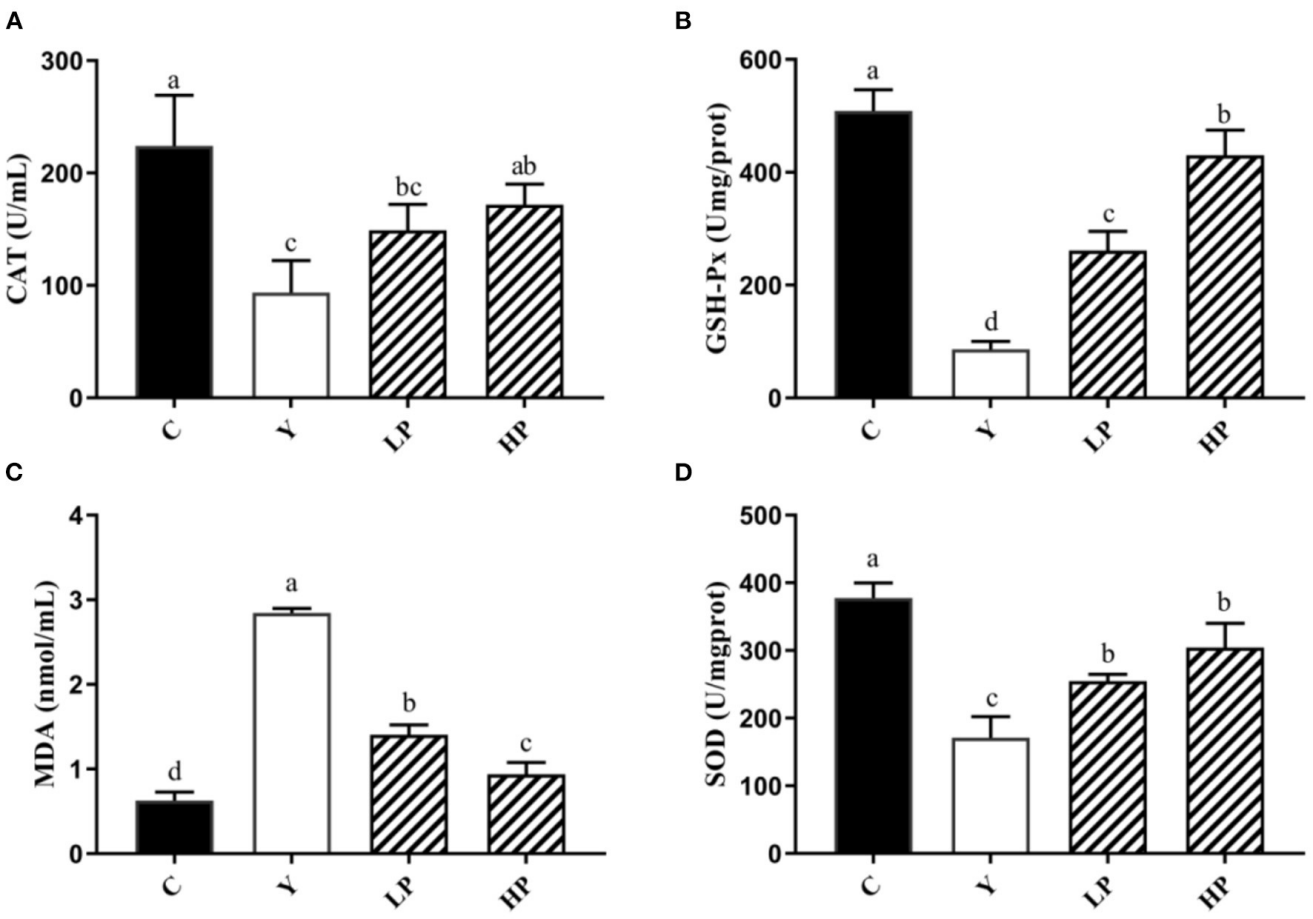
Effects of OP on the contents of CAT **(A)**, GSH-Px **(B)**, MDA **(C)**, and SOD **(D)** in liver of oxidative injured mice. Different letters in the figure represent significant differences (*P* < 0.05). C: the normal group; Y: the Cy-induced group; LP: Cy+200 mg/kg OP; HP: Cy+400 mg/kg OP.

In addition, the dose dependent effects of OP on the activities of the various antioxidant factors in the mouse liver tissue was also analyzed (Figure 5). It was found that compared with Cy treated mice, the activities of CAT, GSH-Px and SOD in liver decreased significantly (*P* < 0.05), while the content of MDA was observed to be increased more than 3 times (*P* < 0.05), thereby suggesting that serious oxidative stress occurred in liver after Cy was injected into the mice. Moreover, Compared with Cy group, CAT, GSH-Px and SOD in the liver of mice increased substantially (*P* < 0.05), and MDA production was decreased approximately by 50% in LP mice and 70% in HP mice (*P* < 0.05) under OP prevention. The above findings indicated that OP also exhibited a specific recovery and regulation effect on oxidative damage caused in the mouse liver tissues, and can display a dose-dependent protective effect.

### Effects of OP on the Relative Expression Levels of the Various Antioxidant Genes in Ileum of Mice

**Figure 6:**
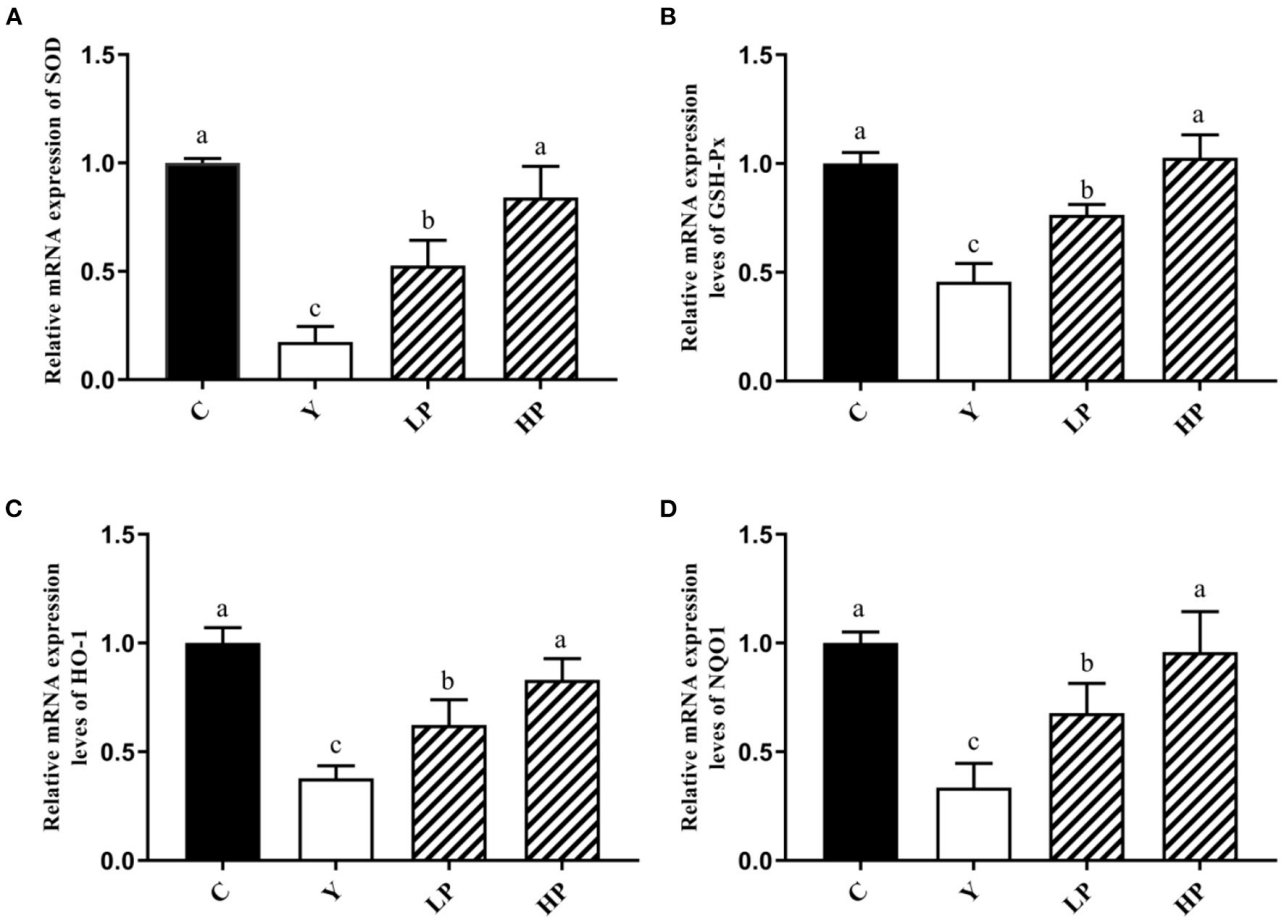
Effects of OP on the relative mRNA expression levels of intestinal antioxidant factors SOD **(A)**, GSH-Px **(B)**, HO-1 **(C)**, and NQO1 **(D)** in oxidative injured mice. Different letters in the figure represent significant differences (*P* < 0.05). C: the normal group; Y: the Cy-induced group; LP: Cy+200 mg/kg OP; HP: Cy+400 mg/kg OP.

The effect of OP on the relative expression of antioxidant related genes in the ileum of Cy-induced mice was also examined. The experimental results have been shown as in Figure 6. It was noted that compared with the normal group, intraperitoneal injection of Cy significantly reduced the relative mRNA expression of SOD, GSH-Px, HO-1 and NQO1 (*P* < 0.05). Moreover, compared with the Cy treated group, the relative expression of SOD, GSH-Px, HO-1 and NQO1 genes were significantly increased upon prior intake of OP (*P* < 0.05) in a dose-dependent manner. These results suggested that OP can regulate the expression of various antioxidant genes and exert substantial protective effect on intestinal oxidation in immunosuppressed mice, which can partially recover the decreased antioxidant capacity caused by Cy.

### Effects of OP on Nrf2-Keap1 Protein Expression in Ileum of Mice

**Figure 7:**
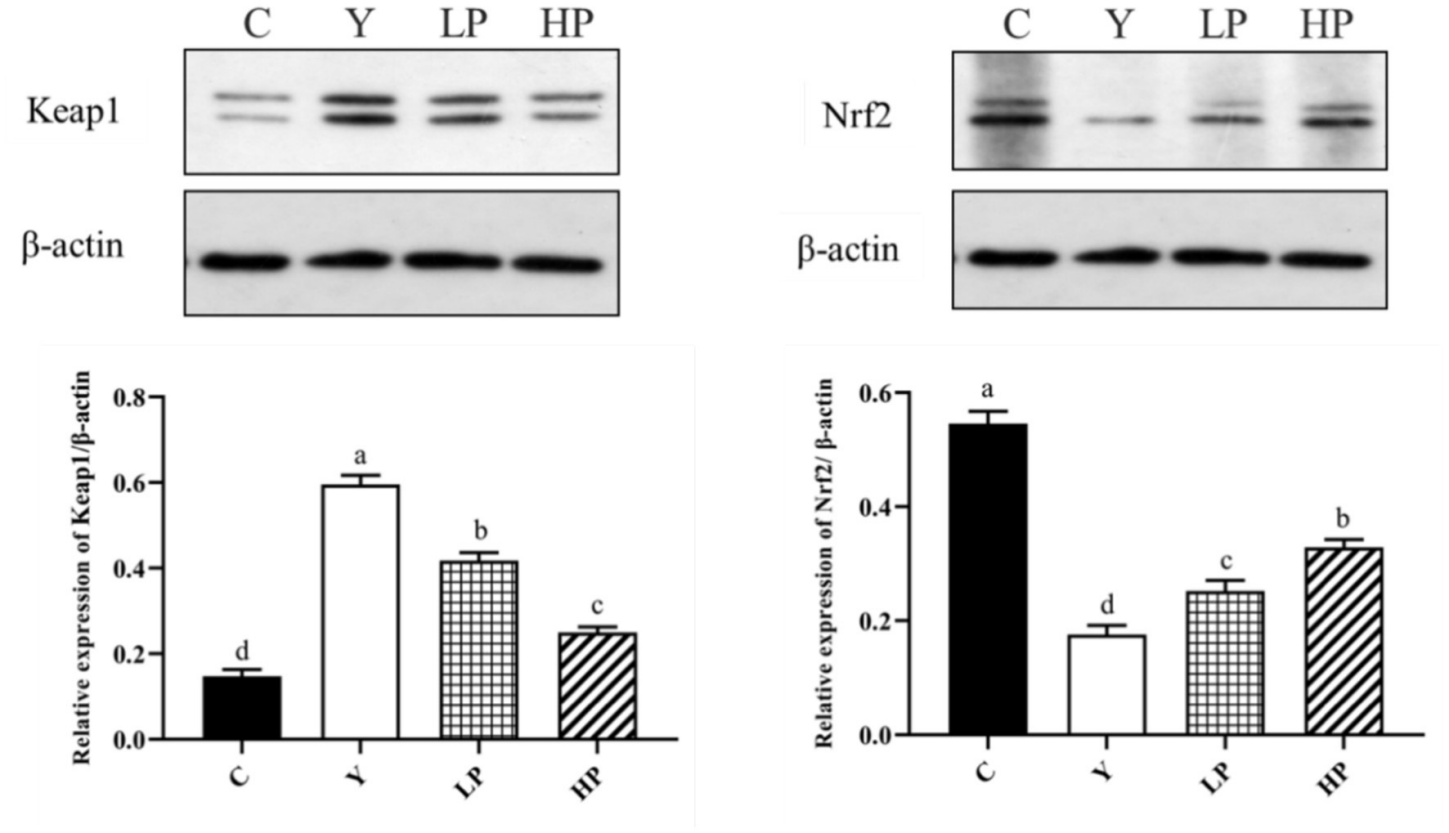
Effects of OP on Keap1-Nrf2 signaling pathway in the ileum of oxidative injured mice. Different letters in the figure represent significant differences (*P* < 0.05). C: the normal group; Y: the Cy-induced group; LP: Cy+200 mg/kg OP; HP: Cy+400 mg/kg OP.

Finally, we investigated the dose dependent effect of OP on the relative expression of major proteins of Nrf2-Keap1 pathway in the mice ileum. The result has been shown in Figure 7. The relative expression level of Nrf2 protein in ileum tissue of mice after Cy treatment was found to be significantly reduced (*P* < 0.05), but the relative expression level of Keap1 protein was significantly increased (*P* < 0.05) as compared with the normal group. Interestingly, prior intake of OP significantly upregulated Nrf2 protein expression level in ileum of the mice (*P* < 0.05), while it significantly decreased Keap1 protein expression in a dose-dependent manner (*P* < 0.05), as compared with the Cy group. These findings suggested that OP might play a protective role on intestinal oxidative stress in immunosuppressed mice through activating Nrf2-Keap1 pathway.

## Discussion

Cy is widely employed in the treatment of different cancers as well as autoimmune diseases (31), and can cause oxidative stress and intestinal mucosal damage while exerting anti-tumor effects (32). It has been established that administration of large doses of Cy can cause severe side effects such as intestinal barrier disruption, increased intestinal permeability, gastrointestinal mucosal destruction, immune deficiency as well as secondary infections, and the production of excessive free oxygen radicals. In recent years, several studies have found that diverse bioactive peptides isolated from the different sources can exhibit strong antioxidant activity (23), which can improve or restore the body's immunity. Solenocera Crassicornis head protein Hydrolysates-Fraction 1 (SCHPS-F1) is mainly composed of different low molecular weight peptides. It has been reported that Cy-induced nephrotoxicity could be alleviated by restoring antioxidant enzyme activity and Nrf2-Keap1 antioxidant pathway-related protein expression. In addition, Fontoura et al. isolated a novel antioxidant peptide from the protease hydrolysates of the feather keratin from corn germ which caused substantial hydroxyl radical scavenging and lipid peroxidation inhibition (33). Here, we investigated the potential protective effects of OP on intestinal mucosal loss and oxidative stress in immunosuppressed mice upon intraperitoneal injection of Cy and pre-gavage administration of OP.

The experimental results showed that most of the mice exhibited decrease in weight, loss of appetite and mental malaise after the treatment with Cy. However, when OP was administered, the appetite began to recover and weight loss in mice was significantly reduced. Intestinal tract not only plays a vital role in nutrient digestion and absorption, but also is the biggest immune organ. The results of HE staining and electron microscopy suggested that the intestinal villi of Cy treated mice was loose, some villi were broken and their structural integrity was destroyed. The villi of LP as well as HP groups were found to be compact, the intestinal injury was improved, and the villi length was close to the normal group. SOD), CAT) and GSH-Px are the key enzymes and regulatory factors involved in the body's antioxidant defense system. For instance, SOD can effectively catalyze superoxide anion and remove free radicals, thereby reducing the lipid peroxidation and maintaining the balance between oxidative and anti-oxidative states (34–36). Meanwhile, GSH-Px and CAT can functionally remove H_2_O_2_ and reduce ·OH level, thus reducing the damage caused by free radicals to the body (37, 38). MDA is the final by-product of endogenous lipid peroxidation, which can lead to DNA rearrangement and even apoptosis (39, 40). Both the ileum and liver are important immune organs of the body. When the intestinal environment is affected by inflammation, oxidative stress and disorder of bacterial community structure, the liver can also produce corresponding adverse reactions. Therefore, the relative expression level of anti-oxidant genes, and the content of antioxidant enzymes in the serum and liver were also measured in this study. The results indicated that the contents of SOD, CAT and GSH-Px in the serum and mice liver were decreased markedly, whereas the content of MDA was substantially increased after the treatment with Cy, thus suggesting that Cy caused oxidative stress in mice. Moreover, compared with Cy treated group, gavage of OP significantly increased the mRNA expression contents of SOD and GSH-Px in the mice ileum, decreased the MDA content and enhanced the secretion of the SOD, CAT, and GSH-Px in the mice serum and liver. These findings suggested that early intake of OP can significantly reduce the lipid peroxidation in the serum, ileum and liver tissues, and alleviate the oxidative lesions induced by Cy. The possible mechanisms contributing to these protective effects may be due to improvement in the contents of antioxidant enzymes by up-regulation of their relative mRNA expression. A number of previous studies have reported many similar results. For example, fucoidan has been shown to significantly reduce the contents of MDA, IL-6, IL-1β, and TNF-α in the liver and kidney tissues of mice induced by Cy (41), and it also increased the activities of SOD, GSH-Px and CAT. In addition, fucoidan can also activate Nrf2/HO-1 pathway, by increasing Nrf2, GCLM, HO-1 and NQO1 protein levels. Sulfate polysaccharide AL1-1 in sea cucumber can significantly increase intestinal total antioxidant capacity (T-AOC) and decrease intestinal MDA level (42). AL1-1 can also increase the levels of CAT and GSH-Px, and significantly enhance the immune response. These studies are consistent with the conclusion of our experiment results.

Nrf2 is a critical transcription factor which can effectively regulate oxidative stress in the body and restore oxidative damage by modulating the secretion of antioxidant enzymes (43, 44). Under regular physiological conditions, Nrf2 remains bound to Keap1 (Kelch-like ECH-associated protein 1), in the cytoplasm, which can also regulate degradation of Nrf2 by the ubiquitin–proteasome pathway. In response to oxidative stress, the continuous production and accumulation of ROS causes Nrf2 to dissociate from Keap1 and migrate into the nucleus to bind to antioxidant response elements (ARE) and regulate the secretion of various cytoprotective genes (HO-1, NQO1) downstream of Nrf2-Keap1 signaling pathway (45). The experimental results showed that intake of OP significantly increased the gene expression levels of major detoxifying enzymes, HO-1 and NQO1 in the Nrf2 signaling pathway of mice ileum. Interestingly, the Western-blot results also indicated that the expression of Keap1 protein in ileum mice induced by Cy was substantially reduced and the expression of Nrf2 protein was up-regulated by OP intake in a dose-dependent manner as compared with the Cy treatment group. Overall, the findings suggest that OP can regulate oxidative stress in Cy treated mice, and the mechanism may be relevant to the activation of pathway of Nrf2-Keap1 to combat the oxidative stress. OP also showed potent antioxidant activity *in vitro*. By regulating Nrf2-Keap1 pathway in CPP-treated mice, the expression of upstream factor Keap1 was decreased, whereas that of Nrf2 was increased, and then the expression of downstream factors (NQO1, HO-1, GSH-Px, SOD and CAT) was found to be enhanced. It prevented oxidative damage induced by Cy in mice. As an effective antioxidant, OP can significantly reduce reproductive oxidative stress damage associated with the Nrf2-Keap1/ARE pathway.

## Study Limitation

This finding may be some potential limitations. First, Cy-induced long-term intestinal oxidative damage is not equivalent to ordinary oxidative damage. The Cy modeling method has indeed increased the success rate and achieved excellent results *in vivo*, but at the same time, it is necessary to ignore the interference effects of other negative effects. In addition, peptides are hydrolyzed in the gastrointestinal tract into free amino acids, which also have the antioxidant capacity in the body. However, it is worth noting that it is also due to the characteristic products of different peptide sequences that cause the different results.

## Conclusion

In this study, intragastric administration of OP was found to significantly increase the average daily gain of Cy-induced oxidative stress mice. OP treatment also improved SOD, CAT and GSHPx activity levels in the serum and liver, and significantly reduced MDA content. Moreover, OP increased the relative mRNA expression of SOD, GSH-Px, Nrf2, HO-1 and NQO1 in ileum significantly, up-regulated Nrf2 protein expression but down-regulated Keap1 protein expression. Overall, OP can regulate the intestinal oxidative damage induced by Cy in mice, and its mechanism may be primarily mediated by enhancing the expression of various antioxidant genes, antioxidant enzyme activities and activating Nrf2-Keap1 pathway. The results showed that OP might have useful potential in the treatment of intestinal oxidative damage and other similar related diseases.

## Data Availability Statement

The original contributions presented in the study are included in the article/supplementary materials, further inquiries can be directed to the corresponding author.

## Ethics Statement

The animal study was reviewed and approved by the Animal Welfare Act and the Guide for the Care and Use of Laboratory Animals, which is approved by the Animal Ethics Committee of Zhejiang University of Technology (20210308038).

## Author Contributions

HC, XX, and SL provided the project administration and funding acquisition. HZ and XZ designed the research and wrote the manuscript. HZ, TL, and QJ executed the experiments and analyzed the data. HC, XX, and YD reviewed and edited this manuscript. All authors have read and agreed to the published version of the manuscript.

## Funding

This work was supported by grants from the National Key Research and Development Program of China (2020YFD0900902) and the National Natural Science Foundation of China (32101947).

## Conflict of Interest

The authors declare that the research was conducted in the absence of any commercial or financial relationships that could be construed as a potential conflict of interest.

## Publisher's Note

All claims expressed in this article are solely those of the authors and do not necessarily represent those of their affiliated organizations, or those of the publisher, the editors and the reviewers. Any product that may be evaluated in this article, or claim that may be made by its manufacturer, is not guaranteed or endorsed by the publisher.
